# Unilateral Lesion of Dopamine Neurons Induces Grooming Asymmetry in the Mouse

**DOI:** 10.1371/journal.pone.0137185

**Published:** 2015-09-23

**Authors:** Assunta Pelosi, Jean-Antoine Girault, Denis Hervé

**Affiliations:** 1 Inserm UMR-S 839, 75005, Paris, France; 2 Institut du Fer à Moulin, 75005, Paris, France; 3 Sorbonne Universités, Université Pierre et Marie Curie (UPMC, Paris-6), Paris, France; Florey Institute of Neuroscience & Mental Health, AUSTRALIA

## Abstract

Grooming behaviour is the most common innate behaviour in animals. In rodents, it consists of sequences of movements organized in four phases, executed symmetrically on both sides of the animal and creating a syntactic chain of behavioural events. The grooming syntax can be altered by stress and novelty, as well as by several mutations and brain lesions. Grooming behaviour is known to be affected by alterations of the dopamine system, including dopamine receptor modulation, dopamine alteration in genetically modified animals, and after brain lesion. While a lot is known about the initiation and syntactic modifications of this refined sequence of movements, effects of unilateral lesion of dopamine neurons are unclear particularly regarding the symmetry of syntactic chains. In the present work we studied grooming in mice unilaterally lesioned in the medial forebrain bundle by 6-hydroxydopamine. We found a reduction in completion of grooming bouts, associated with reduction in number of transitions between grooming phases. The data also revealed the development of asymmetry in grooming behaviour, with reduced tendency to groom the contralateral side to the lesion. Symmetry was recovered following treatment with L-DOPA. Thus, the present work shows that unilateral lesion of dopamine neurons reduces self-grooming behaviour by affecting duration and numbers of events. It produces premature discontinuation of grooming chains but the sequence syntax remains correct. This deficient grooming could be considered as an intrinsic symptom of Parkinson’s disease in animal models and could present some similarities with abnormalities of motor movement sequencing seen in patients. Our study also suggests grooming analysis as an additional method to screen parkinsonism in animal models.

## Introduction

Grooming is the most common behaviour found in mammals, birds and insects [1]. In laboratory rodents it represents up to 30–50% of their waking time [1, 2] and is mainly seen just before and after the diurnal sleep periods [1]. Grooming is considered as a behaviour taking care of the body and fur, but other functions have been proposed. It can contribute to thermoregulation and social relationships, when it is directed not only at one’s own body, but also at that of conspecifics [1]. It can start spontaneously or be elicited by external stimulation such as water spray. Grooming is not a learned behaviour but appears as an innate set of stereotyped movements affecting all parts of body [1]. These movements are often organized in a highly predictable sequence termed “syntactic grooming chain” [2–4]. In rodents, this rigid sequential pattern is characterized by four different phases with rostrocaudal progression. It mainly consists of a series of forelimb movements and licks first targeting the nose (phase 1), then the head (phase 2), and the body flanks (phase 3) until reaching the genitals and tail (phase 4). In normal conditions, the motor sequences are symmetrical, affecting alternatively the left and right side of animals [2–4].

This grooming sequence is organized in a fixed chain of behavioural events that can be considered as a syntax. This stereotyped behaviour is highly sensitive to manipulations, such as pharmacological treatments or gene mutations [5–7]. Striatal function is critical for coordination of grooming sequences. The syntactic disruption of grooming chain occurs particularly when striatal damage extends to a specific region in the dorsolateral striatum, (see [4]). The neurons within this region are absolutely required for implementing the grooming syntax pattern into actual behaviour [4]. Extracellular electrophysiological recordings in the rat dorsolateral striatum have demonstrated that neurons in this region code the entire grooming sequence pattern and that they are especially firing in terminal phases [8]. Those neurons also discriminate between grooming movements within the sequential pattern and outside the syntactic chain [8].

Grooming is regulated by dopamine signalling through activation of D1 and D2 receptors. D1 receptor activation plays a particular role in the grooming accomplishment. D1 receptor agonist (SKF38393) increases the grooming duration and alters the rostrocaudal sequence, by reducing the face grooming and increasing the flank grooming [7]. Moreover, mutant mice lacking D1 receptor are less likely to complete grooming chains than normal mice [9]. When a D2 receptor antagonist, such as haloperidol, is administered prior to D1 agonist the induced grooming increase is prevented, suggesting that D1 receptor-dependent grooming requires endogenous D2 receptor stimulation [10]. Paradoxically, a D2 receptor agonist decreases grooming, probably by acting on D2 autoreceptors [7].

Due to the importance of basal ganglia, and mainly the striatum, in grooming behaviour, it is straightforward to think that diseases affecting this brain circuit, such as Parkinson’s disease (PD) can have effects on grooming. Parkinson’s disease is a progressive neurodegenerative disorder with a loss of nigrostriatal dopamine neurons leading to emergence of tremor, rigidity, akinesia, and bradykinesia. PD is most often asymmetrical, the symptoms being more pronounced in one side early in disease development and this lateralization being preserved throughout the disease [11]. Asymmetries in gait control were also reported in mildly to moderately affected PD patients [12]. The motor asymmetry corresponds to asymmetric loss of dopamine producing cells in the substantia nigra [11]. A previous study has shown that bilateral lesion of dopamine neurons produces alterations in grooming behaviour in the rat, particularly by disrupting the completion of syntactic grooming chains [13]. However, bilateral dopamine depletion induces important aphagia and adypsia in the rodent that often preclude the study of parkinsonism with this approach. A widely used model to study parkinsonism in rodents is the unilateral lesion of dopamine neurons by microinjection of the neurotoxin 6-hydroxydopamine (6-OHDA) into the substantia nigra, striatum or medial forebrain bundle (MFB) [14–16]. This approach leads to severe unilateral dopamine neurons lesions (>80%), and results in alterations in behavioural symmetry characterized by turning on the ipsilateral side of lesion and preferential ipsilateral scanning [17]. They start soon after the lesion and last for several weeks if the lesion is very severe, or recover after 7 days in case of mild lesion [18]. However, the consequences of unilateral 6-OHDA-induced lesion and L-DOPA treatment on grooming behaviour are unclear. In addition, their impact on grooming at ipsilateral and contralateral sides is unknown.

In the current study, we recorded grooming duration and grooming syntactic chain in mice with a unilateral 6-hydroxydopamine lesion, and compared the effects ipsilateral and contralateral to the lesion. The impact of a low dose of L-DOPA on some grooming parameters and asymmetries was also analysed.

## Materials and Methods

### Animals

Thirty-seven 8-week old C57BL/6J male mice were used in two different experiments: 6 sham and 12 lesioned mice in the first experiment and 6 sham and 13 lesioned mice in the second experiment. The mice were maintained in a 12-h light/dark cycle, in stable conditions of temperature (22°C), with access to food and water *ad libitum* and housed 5 per cage. All the experiments were in accordance with the guidelines of the French Agriculture and Forestry Ministry for handling animals (decree 87–848). The laboratory animal facility was approved to carry out animal experiments by the *Sous-Direction de la Protection Sanitaire et de l’Environnement de la Préfecture de Police* (licence B75-05-22 and then *arreté préfectoral* DTPP 2014–724 C75-05-22). The experimental protocols were approved by the *Institut du Fer à Moulin* local review board. The principal investigators had a personal agreement (D Hervé, licence C-75-828; JA Girault, licence 75–877).

### 6-OHDA lesions and postoperative care

Mice were anesthetized with a mixture of xylazine (10 mg/ml) and ketamine (25 mg/ml) (Centravet) and mounted in a digitalized stereotactic frame (Stoelting Europe) equipped with a mouse adaptor. 6-OHDA-HCl (3 mg/ml; Sigma-Aldrich) was dissolved in a solution containing 0.2 g/L ascorbic acid and 9 g/L NaCl. Mice received an intraperitoneal (i.p.) injection of the selective noradrenaline reuptake inhibitor, reboxetine (30 mg/kg; Tocris), 30 min prior unilateral injection [19] of 0.5 μl of 6-OHDA into the right MFB at the following coordinates according to the mouse brain atlas [20]: anteroposterior (AP), -1.2 mm; lateral (L), +1.1 mm; dorsoventral (DV), -5 mm. Each injection was performed with a PCR micropipette (1–10 μl, Drummond Scientific Company) connected to a Narishige system at a slow rate of 0.25 μl/min to minimize tissue damage. After the injection, the micropipette was left in place for 4 additional mins before being slowly retracted. Sham mice were injected with vehicle only (ascorbic acid in saline).

Mice were let on a warm plate during about 24 h after surgery to avoid hypothermia. To reduce suffering, mice received subcutaneous injections of a non-steroidal anti-inflammatory drug (flunixin meglumine, 4 mg/kg; Sigma-Aldrich) just after the surgery and twice daily during the next 2 days. The weakest animals also received injections of 5% glucose (10 ml/kg, s.c.) and saline (10 ml/kg, i.p.) to avoid dehydration, until they reached 80% of their initial weight. Sweetened condensed milk was provided to all the lesioned animals during one week after the operation. Mice were allowed to recover for 3 weeks before behavioural evaluation and 4 weeks before drug treatment. Lesions were assessed at the end of experiments by determining the striatal levels of tyrosine hydroxylase (TH) using immunoblotting (see below). Only animals with a TH level reduction by more than 70% in the lesioned striatal area compared with the control side were included in the analyses.

### L-DOPA and benserazide treatments

Mice were injected with L-DOPA 4 weeks after the lesion. L-DOPA and the peripheral DOPA decarboxylase inhibitor benserazide hydrochloride (Sigma-Aldrich) were dissolved together in physiological saline solution (9 g/L NaCl) and i.p. injected at a dose of 2.5 and 12 mg/kg, respectively, in a volume of 10 ml/kg body weight as acute treatment (one injection).

### Analysis of grooming and other behaviours

Prior to testing, mice were transported from their holding room to the testing room and allowed at least 1 h for acclimation. All observations were performed between 2:00 and 7:00 pm to ensure uniformity throughout trials. Animals were individually placed in a clear cylinder (13 cm in diameter, 16 cm height) for behavioural observation. To assess spontaneous grooming, the mice were video-recorded for 2–3 h using the HomeCageScan software (CleverSys, Inc., Reston, VA) by side-view and top-view web cameras (Pacific Electro-optics model PA-290 DN). Some mice were also video-recorded for 3 h after L-DOPA injection. The observation cylinder was thoroughly cleaned using 30% ethanol (vol/vol) between subjects.

The videos were manually analysed as described by Kalueff et al. [2]. During manual scoring, a highly trained observer blinded to the group, used the Grooming Analysis Algorithm (GAA) [3] to record the latency, direction and duration of each grooming bout and its constitutive episodes (paw licks, head washes, body/leg washes and tail/genital washes), as described previously [2, 6]. A grooming “bout” was characterized as continuous self-grooming without interruption (defined as a full stop in grooming action for more than 3 s). An “episode” was identified as a portion of a single bout in which the subject is grooming a specific body region (e.g., paw licks and body/leg washes), lasting less than 5 s, and a “transition” was defined as a progression from one grooming episode to another separate episode within a single grooming bout, according to Kyzar et al. [6]. Single grooming duration was calculated as total duration divided by the number of bouts [2].

The number of vertical rears and sleeping time (“resting time”) during the observation session were also scored for each mouse. The number of clockwise and counter-clockwise rotations (360°) made by the animals were also counted during the sessions.

Data were generated for the percentage of ipsilateral events for turns and grooming, compared to the total events, the percentage of complete vs. incomplete grooming bouts and for the percentage of correct vs. total transitions. A correct transition was defined following the typical cephalo-caudal progression (i.e., paw lick—phase 1 > head wash—phase 2 > body/leg wash—phase 3 > tail/genital grooming—phase 4), see details in [3].

### Immunoblotting

**Fig 1:**
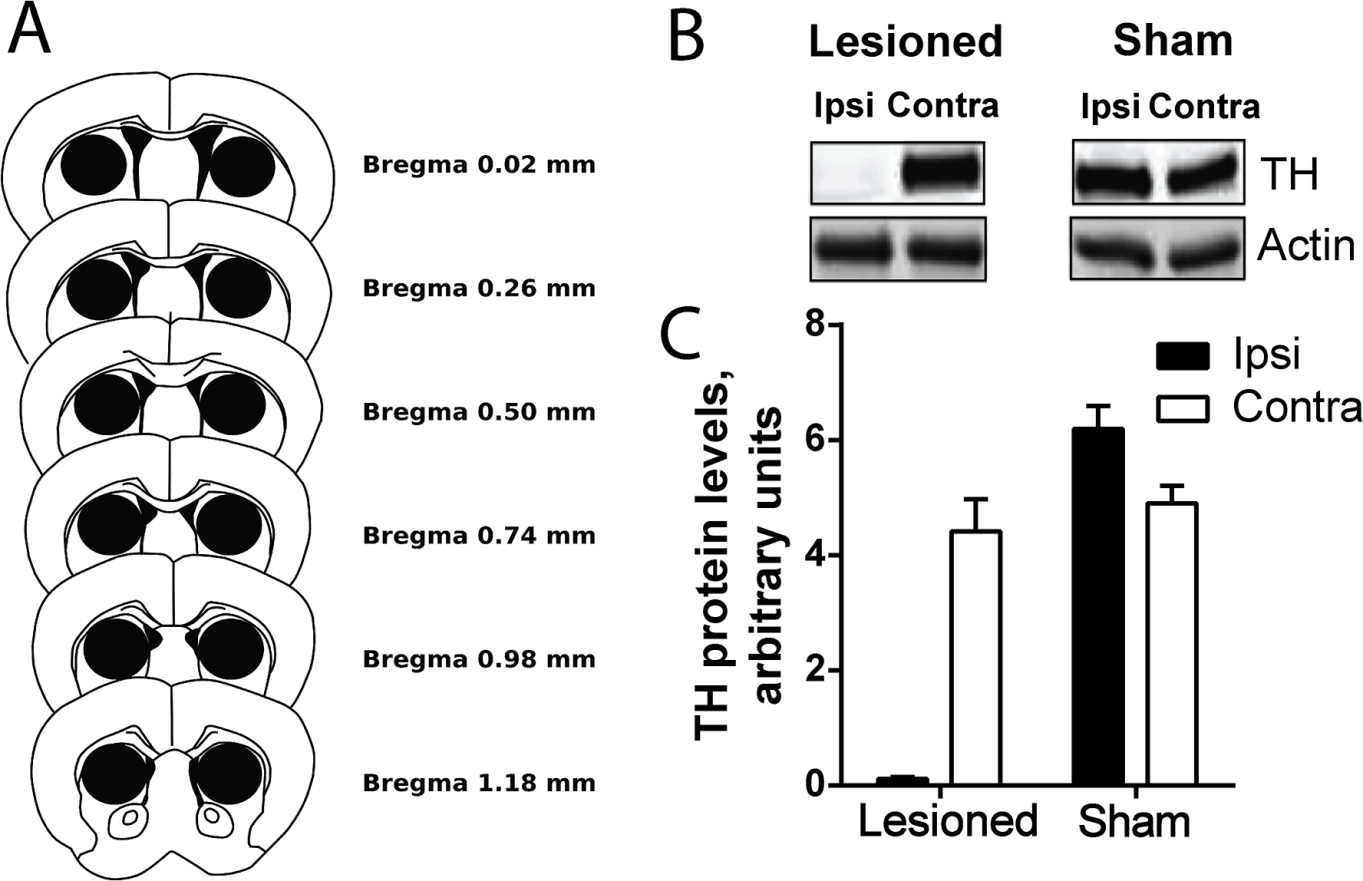
Lesion severity. **(A)** Schematic representation of brain sections showing where the tissue samples were taken out from striatal slices. **(B**) Example of immunoblot of tyrosine hydroxylase (**TH**) in 6-OHDA-injected and sham animals. Actin was measured as loading control. (**C)** Quantification of TH in immunoblots as in B in animals used for behavioural studies (lesioned n = 18, and sham n = 12).

Sham-operated and 6-OHDA-injected mice were killed by decapitation and their heads were immediately frozen in liquid nitrogen (12 s) [21]. The frozen heads were then sliced with a cryostat (210-μm-thick) and six microdisks (1.4-mm diameter) were punched bilaterally from the dorsal striatum (Fig 1A) and stored at -80°C. The samples were sonicated in 10 g/L sodium dodecyl sulphate (SDS), and placed at 100°C for 5 min. Aliquots (5 μl) of the homogenate were used for measuring protein concentration using a bicinchoninic acid assay kit (Pierce Europe). Equal amounts of protein (20 μg) were separated by 15–10% (w/v) pre-casted gel (Bio-Rad) in the presence of SDS and transferred to nitrocellulose membranes (GE Healthcare) [22].

The membranes were immunoblotted using TH monoclonal mouse (Millipore) and actin monoclonal rabbit (Sigma) antibodies and IRDye 800CW-conjugated anti-mouse and anti-Rabbit IgG (1:5000) (Rockland Immunochemical) as secondary antibodies. Their binding was quantified using an Odyssey–LI-COR infrared fluorescent detection system (LI-COR).

### Statistics

Multiple comparisons were analysed using one-way ANOVA, followed by Sidak’s *post hoc* tests for multiple comparisons. For two-group comparisons, two-tailed unpaired Student’s *t* test with Welch’s correction was used. Normality was assessed by Kolmogorov-Smirnov test with Dallal-Wilkinsons-Lieliefor P Value (Graph Prism 6).

## Results

### Lesion in the medial forebrain bundle induces reduction and asymmetry in grooming

Three weeks after 6-OHDA or vehicle injection in the MFB, the mice were video-recorded to study self-grooming behaviour. At the end of experiments, the TH levels were measured in the striatum of sham-operated and 6-OHDA-injected animals (Fig 1). Seven 6-OHDA-injected mice were excluded from the analysis because they were not well lesioned (TH levels above 30% of the control levels). The remaining 18 correctly lesioned animals were used to score grooming.

**Fig 2:**
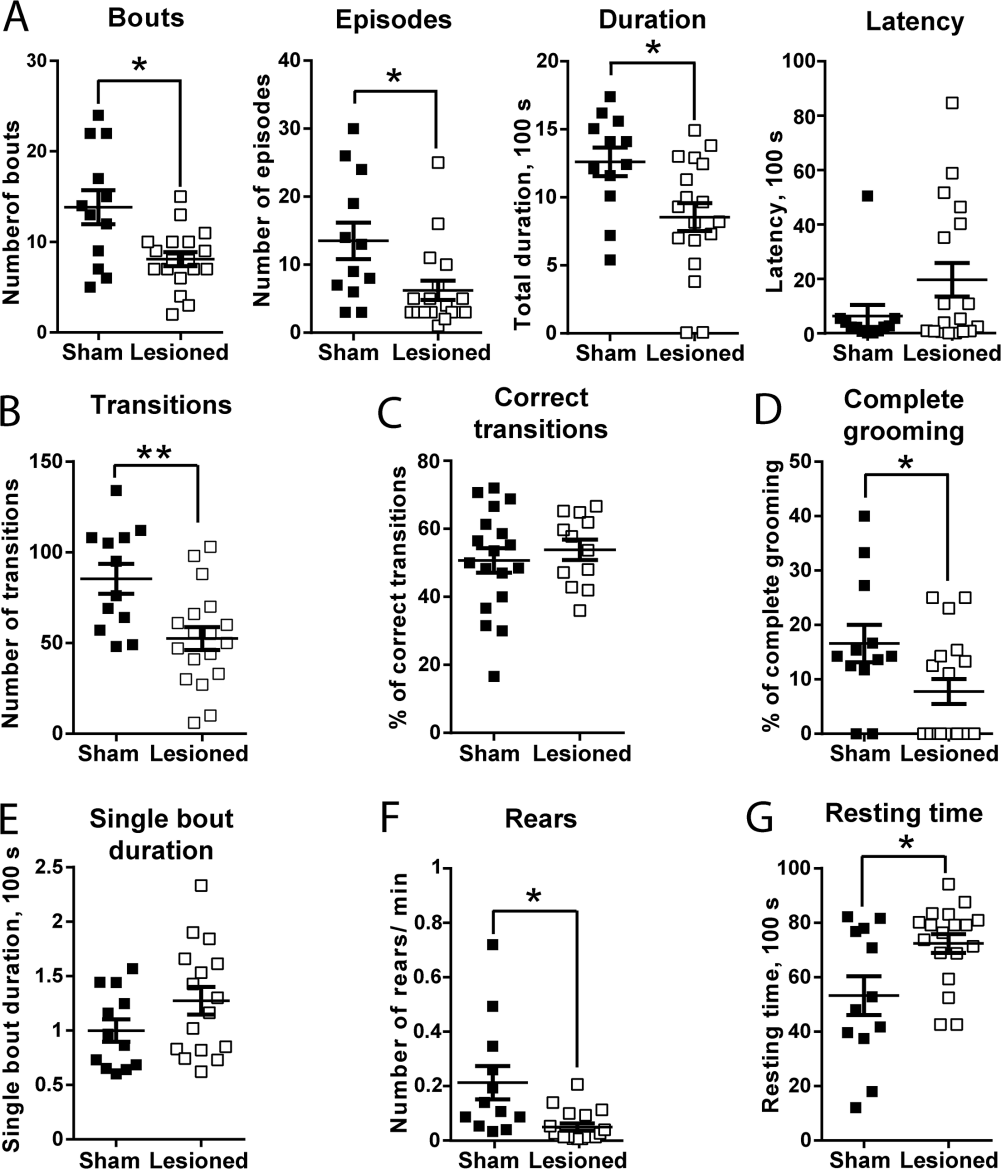
Effects of unilateral 6-OHDA lesion on grooming behaviour. Analysis was done in mice 3 weeks after a unilateral injection of vehicle (sham, n = 12) or 6-OHDA (lesioned, n = 18) in the MFB. Data are analysed using unpaired two-tailed t test with Welch’s correction. (**A)** Analysis of bouts and episodes: bouts, t = 2.83, p < 0.05, episodes, t = 2.41, p<0.05, total time spent in grooming behaviour (duration), t = 2.78, p < 0.01, latency, t = 1.81, p = 0.08. (**B-D)** Analysis of the four main phases of grooming chain: total number of transitions, t = 3.17, p < 0.01 (B), percentage of correct transitions, t = 0.66, p = 0.51 **(C)**, percentage of complete grooming bouts, t = 2.16, p < 0.05 (D). (**E)** Duration of single bouts, t = 0.62, p = 0.53. (**F, G)** Analysis of non-grooming behaviour. Number of rears per minute, t = 2.60, p<0.05 (F). Resting time, t = 2.43, p<0.05 (G). *p< 0.05, **p< 0.01.

Using manual analysis, we found that grooming behaviour was decreased in unilaterally 6-OHDA-lesioned mice as compared to sham-operated animals. The number of bouts and episodes (see Materials and Methods) was reduced (Fig 2A). Overall, there was a reduction in the total time the animals were spending in grooming behaviour (Fig 2A). A longer latency for grooming was found in some animals, but was not significant overall (Fig 2A). Analysis of the four main phases of grooming chain (Fig 2B–2D) showed a decrease in total number of transitions by more than 50% as compared to sham animals (Fig 2B), without any change in the percentage of correct transitions (Fig 2C), showing that the general structure of grooming behaviour (syntax) was not affected in lesioned animals. However, the percentage of complete grooming bouts in which all four phases of the grooming chain were present was decreased (Fig 2D). The duration of single bouts was not significantly different between lesioned and sham animals (Fig 2E), demonstrating that the reduced time spent in grooming was a result of reduced frequency of grooming events. A significant difference in frequency of rears and resting time was also found (Fig 2F and 2G, respectively), indicating a reduced general activity in the lesioned group as compared to the control group.

**Fig 3:**
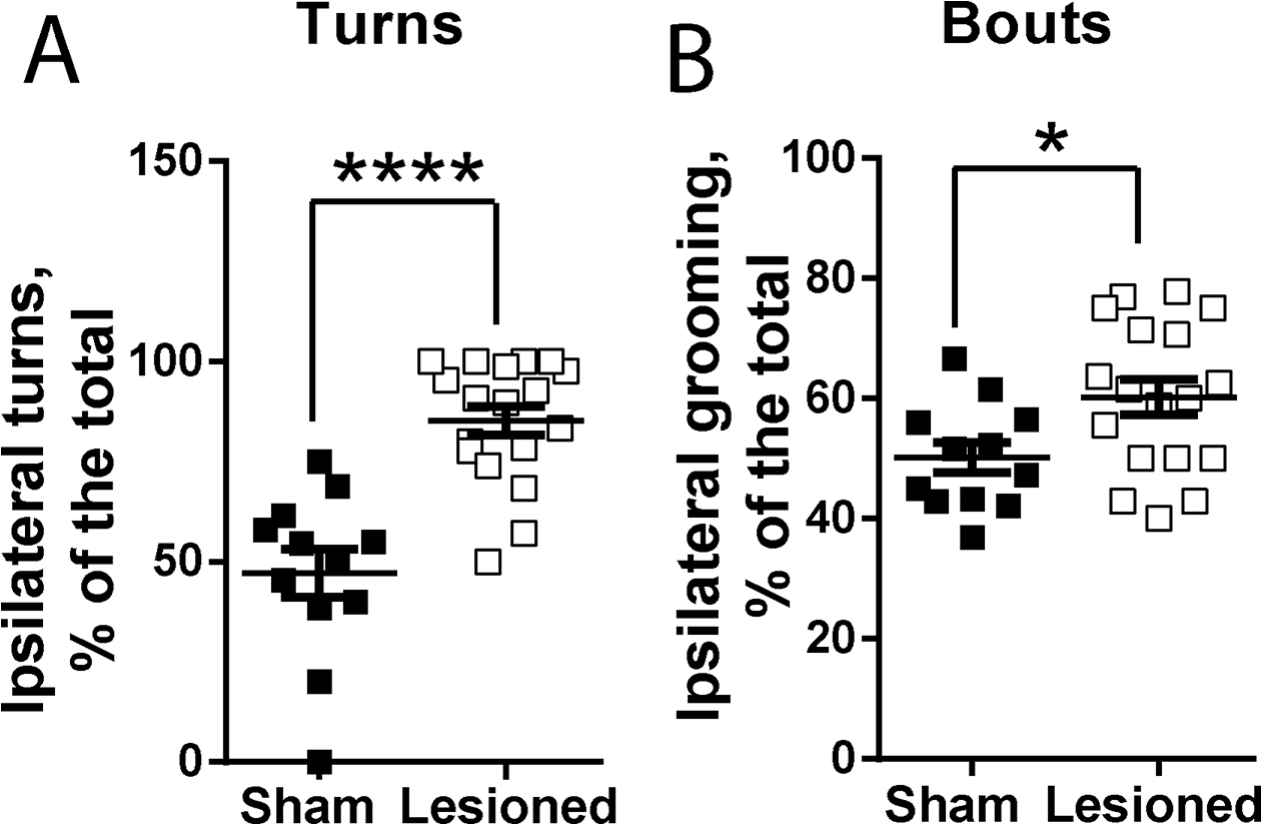
Analysis of behavioral asymmetries. Analysis was done in mice 3 weeks after a unilateral injection of vehicle (sham, n = 12) or 6-OHDA (lesioned, n = 18) in the MFB. Data are analysed using unpaired two-tailed t test with Welch’s correction. (**A)** Ipsilateral turns, calculated as % of the total turns t = 5.42, p < 0.0001. (**B)** Ipsilateral grooming as a % of total grooming, t = 2.60, p = 0.014. *p< 0.05, **** p< 0.0001.

As compared to sham animals, we observed in 6-OHDA-lesioned mice significant behavioural asymmetries. They were characterized by a rotation behaviour towards the side of lesion (ipsilateral side, right in our experiments) (Fig 3A), as reported by Ungerstedt and colleagues [14]. We also observed asymmetries in self-grooming, since the lesioned animals displayed significantly more grooming bouts on the ipsilateral side compared to the total grooming events (directed both to the ipsilateral and contralateral sides to the lesion) (Fig 3B). Sham animals did not show any asymmetry.

Together, these results demonstrated that unilateral lesion of dopamine neurons induced changes in self-grooming behaviour, not only affecting duration and numbers of events, but also breaking the grooming chains as evidenced by the reduced number of transitions and percentage of complete grooming chains. However, the syntax of grooming events appeared to be preserved since the percentage of correct transitions was not affected by the lesion. The unilateral disruption of nigrostriatal pathway altered the characteristic symmetry of this behaviour by producing reduced contralateral grooming.

### Acute injection of low dose of L-DOPA restores the grooming behaviour

To test the possibility that L-DOPA at low dose (2.5 mg/kg) could have a positive effect on the grooming changes and asymmetries developed after surgery, a new cohort of animals was used. Before performing the surgery, animals were recorded for 3 h in the same conditions used in the previous experiment to analyse the basal level of grooming. Mice were video-recorded again 3 weeks after lesions to analyse asymmetries in rotation and grooming as well as other grooming parameters. Four weeks after surgery both groups of animals (8 lesioned and 6 sham animals) were treated once with 2.5 mg/kg of L-DOPA (associated with 12 mg/kg of benserazide), a dose that produced few abnormal involuntary movements characteristic of LID. To avoid that LID interferes with grooming analysis, grooming and rotation behaviours were analysed between 1.5 and 3 h after L-DOPA injection.

**Fig 4:**
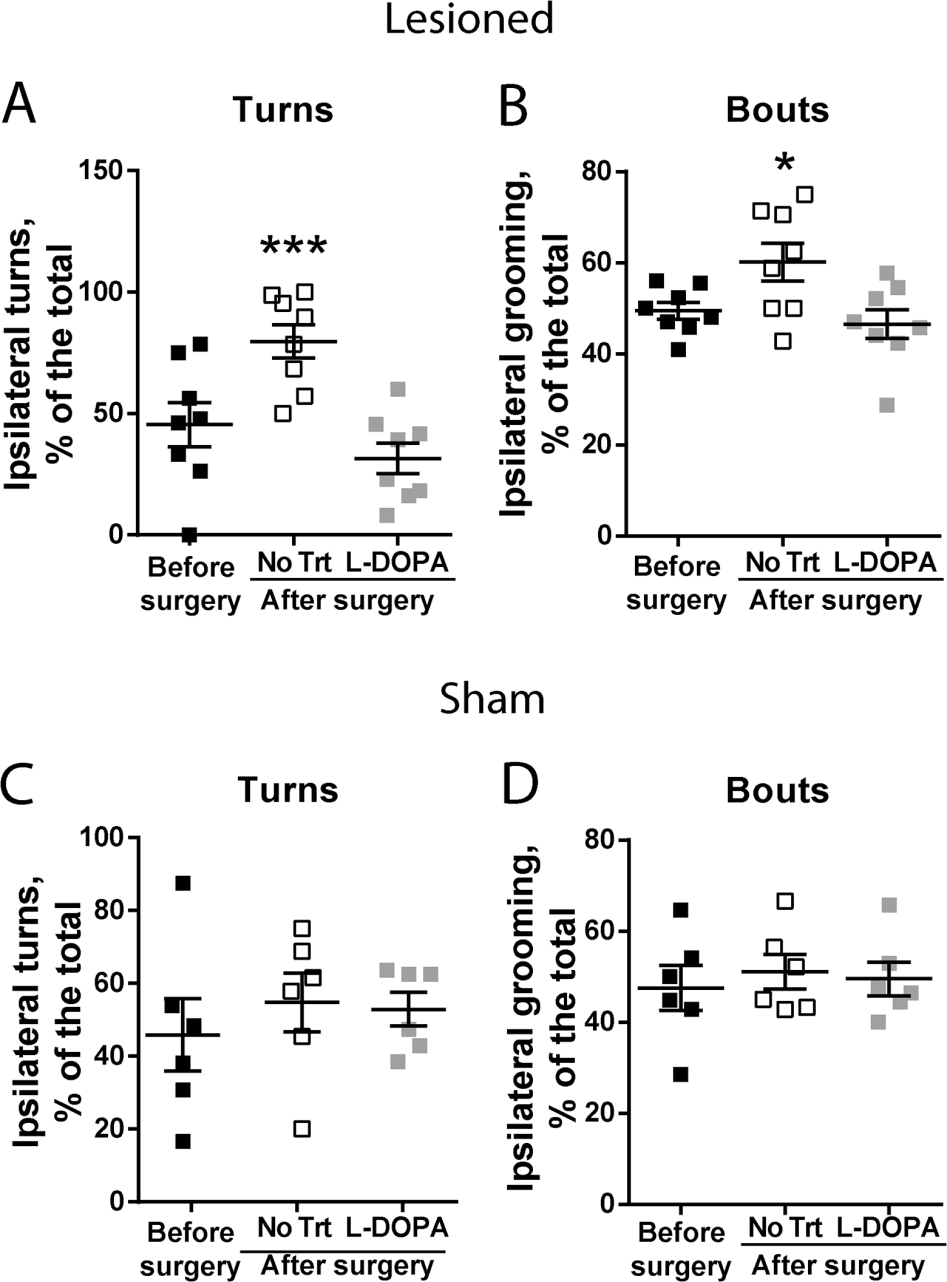
Analysis of asymmetry of grooming activity. Analysis was done in mice which received a unilateral injection of 6-OHDA (A and B, lesioned, n = 8) or vehicle (C and D, sham, n = 6) into the MFB. Mice were video-recorded before the surgery, 3 weeks after the surgery in the absence of treatment (After surgery, No Trt), and 4 weeks after the surgery, between 1.5 and 3 h after the injection of 2.5 mg/kg L-DOPA and 12 mg/kg benserazide (After surgery, L-DOPA). (**A)** Ipsilateral turns, calculated as % of total turns in the lesioned mice, F_(2,21)_ = 10.9, p < 0.001. (**B)** Ipsilateral grooming, as a % of total grooming bouts in the lesioned animals, F_(2,14)_ = 4.27, p < 0.035. (**C)** Ipsilateral turns, calculated as % of total turns in the sham-operated mice, F_(2,10)_ = 0.56, p = 0.58. (**D)** Ipsilateral grooming, as a % of total grooming bouts in the sham-operated mice, F_(2,15)_ = 0.18, p = 0.83. Statistical analysis, one-way ANOVA followed by Sidak’s post-hoc tests, *p < 0.05, ***p < 0.001.

Asymmetries in turning and grooming behaviours developed after the lesion, as compared to the analysis prior to surgery (Fig 4A and 4B). L-DOPA treatment restored normal symmetrical activities for both turning and grooming behaviours (Fig 4A and 4B). No change in any condition was observed in the sham-operated mice (Fig 4C and 4D).

**Fig 5:**
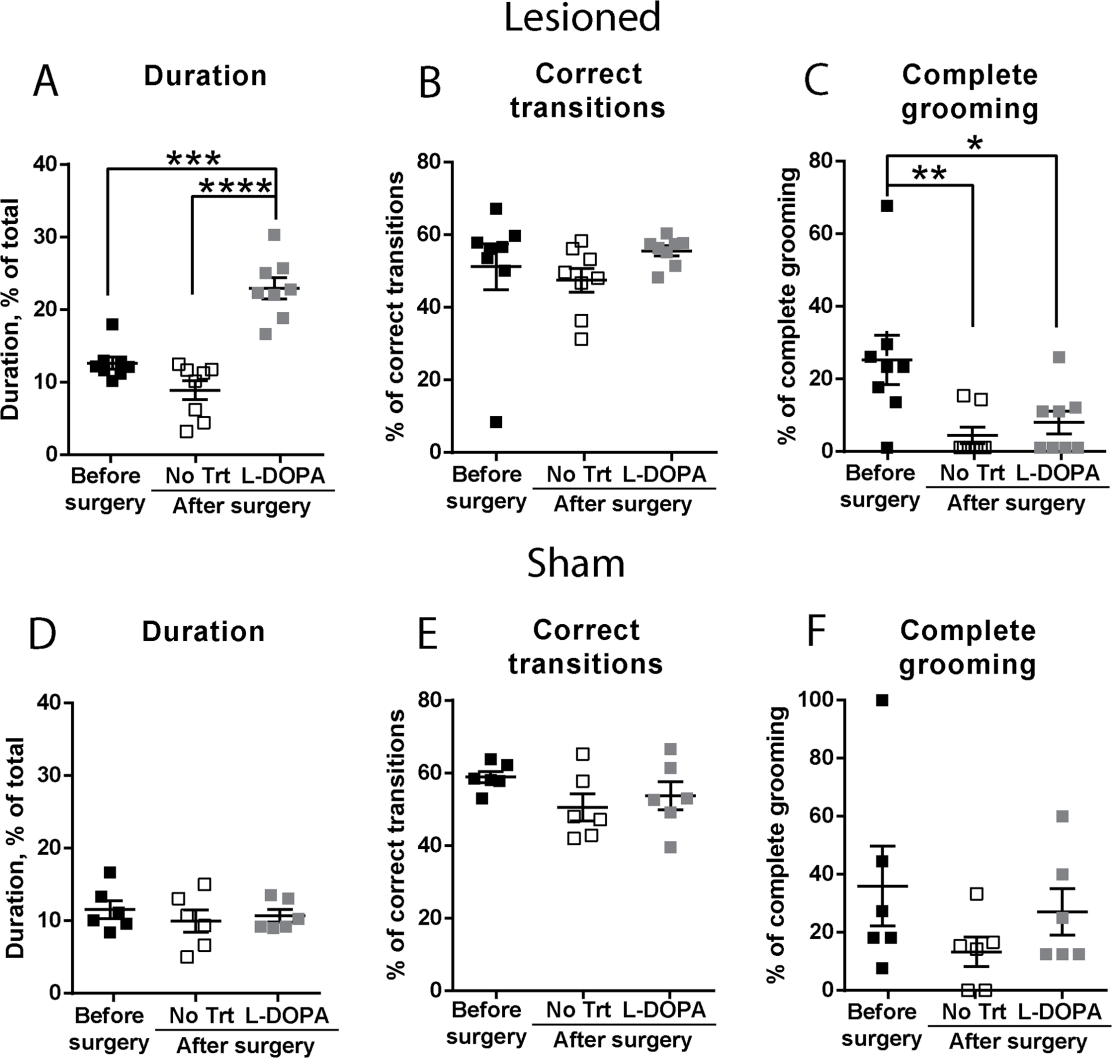
Effects of L-DOPA on grooming behaviour. Analysis was done in mice which received a unilateral injection of 6-OHDA (A, B, and C) or vehicle (D, E and F) into the MFB, as in Fig 4. Mice were video-recorded before surgery and after surgery without treatment (No Trt) and following L-DOPA administration (L-DOPA), as described in the legend to Fig 4. Statistical analysis was done using one-way ANOVA with Sidack’s correction for multiple comparisons. (**A)** F_(2,21)_ = 33.7, p = 0.0001. (**B)** F_(2,21)_ = 1.04, p = 0.29. (**C)** F_(2,21)_ = 6.01, p = 0.025. **(D)** F_(2,17)_ = 0.41, p = 0.62. **(E)** F_(2,17)_ = 1.83, p = 0.21. **(F)** F_(2,17)_ = 1.12, p = 0.35. *p< 0.05, **p< 0.01, ***p< 0.001, ****p<0.0001.

L-DOPA-treated animals showed a general increase in the total duration of grooming (Fig 5A), compared to that observed before and after surgery without L-DOPA treatment. However, in these treated mice, we detected no significant change in the frequency of correct transition (Fig 5B). In addition, the grooming behaviour was stopped prematurely in L-DOPA-treated lesioned mice as it was observed in lesioned mice without L-DOPA treatment. In fact, after L-DOPA treatment, the frequency of complete grooming did not increase as compared to untreated lesioned mice and remained below the value obtained before lesion (Fig 5C). Sham-lesioned animals did not respond to L-DOPA treatment, as shown in Fig 5D–5F. Altogether, these data showed that L-DOPA treatment was able to reduce asymmetries due to the lesion. In addition, L-DOPA treatment increased grooming duration, but without increasing the completion of full grooming sequences. These effects could be linked to general hyperactivity since increase in grooming activity was parallel with an increase in the time animals spent in other kinds of behaviours, such as rearing (data not shown).

## Discussion

In rodents grooming is a natural behaviour characterized by a complex pattern of actions, forming a syntax chain [23,24] Grooming has been extensively studied to elucidate brain regions involved in its regulation and more recently to analyse phenotypes following mutations and manipulations in animal models, especially for anxiety studies [6]. The present study demonstrates that unilateral 6-OHDA lesion in the MFB reduces the self-grooming behaviour of mice. Reduction in self-grooming concerned the overall grooming activity in terms of time and bout number. Decrease was also found in total number of transitions and percentage of complete grooming. Importantly this work shows development of grooming asymmetry directed to the ipsilateral side to the lesion.

Although major findings about grooming features resulted from studies of the effects of lesions in various brain regions, to our knowledge, none reported changes in grooming syntax following unilateral 6-OHDA-induced lesions as a model for hemiparkinsonism. Moreover, the effects of this lesion on grooming asymmetries were not investigated. One study in 6-OHDA-lesioned rats reported intense grooming directed toward the contralateral side to the lesion following treatments with various dopamine agonists but did not describe grooming deficits in untreated animals [25]. Our study tested whether grooming could be an informative behavioural analysis, about the effects of unilateral 6-OHDA lesion in mice. We found a decrease in number of bouts and episodes as well as in time spent grooming. Our analysis also focused on the execution of grooming sequences. In each bout we counted the total number of transitions between one phase and the other, the correct sequence of transitions (syntax) and the percentage of complete vs. incomplete grooming bouts. The results showed a decrease in total number of transitions and complete grooming sequences, as reported following bilateral lesion of substantia nigra [13]. However, when the transitions were executed, they were correct, displaying the characteristic rostrocaudal sequence. In addition, an overall reduction in grooming activity duration was observed, in contrast with the results reported in rats with bilateral 6-OHDA lesion, in which the total amount of grooming was not affected [13]. This discrepancy could be explained considering the different techniques for inducing grooming in this study and ours. After bilateral lesion, grooming was elicited by spray with light water mist, while in our case the animals were habituated to the observation environment prior to start the recording. Moreover, in our experiments, the analysis lasted for longer time, to avoid the grooming activity induced by stress or novelty. It is possible, that in the period that we considered as habituation, during which novelty-induced grooming would arise, lesioned animals were not showing significant difference in the grooming amount and duration. We cannot either exclude that the discrepancy results from differences between mouse and rat. The reduction of grooming activity is also in contrast with results reported by Fornaguera et al. [18]. These authors did not find any correlation between 6-OHDA unilateral lesion severity and duration of grooming since, in their experiments, animals with severe and partial lesions of dopamine neurons displayed similar behaviour. However, it was unclear in these experiments whether grooming behaviour was altered in lesioned animals when compared to unlesioned rats.

Despite the general reduction in grooming activity, no change was found in the single bout duration, suggesting that each grooming phase was lasting longer and syntactic grooming chain was more frequently interrupted before completion. This is consistent with the reduction in number of transitions and rate of complete grooming observed in 6-OHDA lesioned animals. These results are also in agreement with those found after bilateral lesion of nigrostriatal neurons [13]. In this study, the lesioned animals were shown to maintain their capacity to produce grooming actions, but lose their ability to complete the sequences until the end of syntactic chains. It was shown that the rate of completion of action chains was dramatically impaired by excitotoxic lesion of anterior laterodorsal part of striatum [4]. In this region, neurons fire preferentially when the grooming movements are organised in sequence compared with isolated grooming movements or behavioural resting [8]. These data support a specific role of anterior dorsolateral striatum in the control of sequential organization of grooming movements. Following unilateral 6-OHDA lesion, we observed a significant reduction in sequence completion rate, albeit less drastic than that observed after excitotoxic lesion of dorsolateral striatum [4]. Altogether these results suggest that dopamine innervation of the anterior dorsolateral striatum controls the neuronal activity necessary for complete sequences of grooming movements.

We also found a development of ipsilateral preference in grooming syntax execution, resulting probably from a deficit in grooming behaviour on the contralateral side to the lesion. Behavioural asymmetries after lesion were already found to occur for turning and scanning behaviours, but not for grooming [17, 26]. Asymmetries of motor symptoms reflect the degeneration of dopamine neurons, which is contralateral to the more affected side [27]. In our animals, the lesion was performed in the right MFB and animals showed less grooming on the left side, resulting in an increase in the percentage of ipsilateral movements. Lateralization of movement deficits makes PD an asymmetric disease. In view of that, we suggest that evaluation of grooming asymmetries in unilaterally lesioned rodents can, together with turning and scanning, be included in the battery of behavioural tests to analyse parkinsonism in rodents. Abnormal asymmetry was reduced by administration of a single low dose of L-DOPA while no effect was found in sham animals.

L-DOPA administration also had effects on other grooming parameters. The main effect was a general increase in total grooming duration, an effect that could be related to the increase of general activity stimulated by L-DOPA [28]. In addition, we did not observe any L-DOPA effect on the frequency of complete grooming, suggesting a lack of efficiency of L-DOPA to restore sequence completion that was altered by the 6-OHDA lesion. It suggests that dopamine alone cannot re-establish the normal activity controlling the implementation of sequences. It is likely that the presence of fully regulated dopamine neurons is required to generate full chains of grooming movements.

Simultaneous activation of both D1 and D2 receptors at postsynaptic levels can explain the hyperactivity [29] and changes in self-grooming duration. In rats, D1 agonist provoked an increase in grooming duration and preference of grooming towards body flanks [7]. D2 agonist, on the contrary, reduced the duration of grooming, by decreasing extracellular dopamine levels in the striatum through an action on the pre-synaptic D2 receptor [7, 30]. In addition, after D2 agonist treatment, the grooming was directed at the genital region [7] Stimulation of D1 receptor by agonist can also increase stereotypies [8]. In animals treated with D1 agonist, the grooming duration increases mainly because the animals once the grooming bout started, cannot end it before completing all the syntactic sequence [10]. At the same time, D1 agonist caused a decrease in the duration of fixed action pattern, increasing the number of transitions per grooming event [31]. A recent study [32] has shown that the direct pathway (D1 receptor) is mainly active during the start and stop actions of motor sequences, while the indirect pathway (D2 receptor) is more active during the execution of sequences, supporting the theory of organization of learned motor information in chunks [33]. Accordingly, we can hypothesize that after lesion, the reduced activation of the direct pathway will affect the initiation of grooming bouts and the end of syntactic chains, as we found in our animals (reduced number of bouts and episodes and increase of incomplete grooming events). At the same time, alteration of the indirect pathway, mainly involved in the execution of the sequence, will affect the phase transitions. In consistency with this, we found a reduction in the grooming duration, increases in incomplete bouts and in the total number of transitions. In fact, in a grooming study, in which D1 and D2 receptors were stimulated by agonists, it was possible to attribute to D1 stimulation a role in initiation of grooming bouts, and to D2 activation, a role in the modulation and implementation of grooming sequences [7].

The fact that self-grooming is an innate motor behaviour and the grooming syntax pattern is conserved among mammals suggests that the components of neural circuits supporting this behaviour have been optimized during evolution. In addition, it is possible that the brain structures initially specialized to carry out grooming, were later diverted to other kinds of elaborated behaviours such as speech or complex sequence learning. For example, mutation of FoxP2 which is responsible for speech deficits in human produces impairment in motor learning in mice [34]. Language deficit in PD patients, such as increase in syllable repetitions while speaking, have been related to predominant reduction of dopamine in the left hemisphere [35]. Thus, we can speculate that alteration of dopamine availability in the striatum or its asymmetric concentration [35, 36] may have similar consequences on grooming in rodents and some aspects of speech in humans.

### Conclusions

Our findings indicate that analysis of self-grooming activity can be a novel behavioural test to evaluate parkinsonian behaviour in animals with a lesion of dopamine neurons. In addition they demonstrate that a low dose of L-DOPA is able to rescue alterations of grooming symmetry. Finally, they show that sequence completion is affected in unilaterally lesioned animals as in those in which the lesion of dopamine neurons was bilateral [13] or those in which the neurons of anterior dorsolateral striatum were destroyed [8]. These data can help to understand the mechanisms involved in behavioural rigidity and asymmetries found in PD patients, in which action sequence, such as speech, walking or writing, are executed with difficulty and most of the time with one side of the body more affected [35–38]. Our data can also be useful from an experimental point of view, because grooming analysis can improve evaluation of parkinsonian symptoms in animal models.
